# Ki67 and TNFRII as Potential Clinical Markers for Effective Clinical Staging of Advanced Prostate Cancer

**DOI:** 10.3390/cancers17162638

**Published:** 2025-08-13

**Authors:** Edyta Idalia Wolny-Rokicka, Marta Grabowska

**Affiliations:** Department of Histology and Developmental Biology, Faculty of Health Sciences, Pomeranian Medical University, Żołnierska 48, 71-210 Szczecin, Poland; edyta.wolny@gmail.com

**Keywords:** prostate cancer, palliative radiotherapy, prostatectomy, Ki67, apoptosis, inflammation, tumor necrosis factor receptor 2

## Abstract

**Background**: Currently, it is a priority to develop prognostic biomarkers that would allow the identification of patients with progressing prostate diseases with a low risk of progression, so that unnecessary treatment and patient burden can be avoided. **Objectives**: This study aimed to assess the clinical features and concentrations of selected mediators of apoptosis, markers of inflammation, and immunoexpression of Ki67 and selected mediators of inflammation in patients with PCa after prostatectomy procedures and who underwent palliative radiotherapy for bone metastases, as well as patients with benign prostatic hyperplasia (BPH). **Methods**: A total of 88 patients, including 54 cases with PCa and 34 with BPH, were included. Stage and tumor grades were determined according to Gleason’s grading system. Immunoenzymatic methods were used to determine apoptotic and inflammatory mediators in serum, as well as deoxynucleotidyl transferase-mediated dUTP Nick End Labeling (TUNEL) reaction, and immunohistochemistry to determine selected markers of inflammation and Ki67 expression. **Results**: Strong differentiating indicators were revealed, such as Ki67, and tumor necrosis factor receptor 2. Ki-67 expression was significantly associated with the Gleason score. In turn, the tumor necrosis factor receptor 2 (TNFRII) showed the highest expression in the inflammatory environment of cancer in the metastatic stage. **Conclusions**: Ki67 and TNFRII can be classified as high-value clinical markers. These factors may be treated as markers regarding future information about the implementation or withdrawal of more aggressive treatment to possibly avoid toxic complications associated with both increased doses in radiotherapy and prolonged hormonal therapy.

## Simple Summary

Currently, it is a priority to develop prognostic biomarkers that would allow the identification of patients with progressing prostate diseases with a low risk of progression, so that unnecessary treatment and patient burden can be avoided. Evaluation of the selected mediators of apoptosis and markers of inflammation in patients with prostate cancer (PCa) after prostatectomy procedures and undergoing palliative radiotherapy for bone metastases may allow for separate factors that can be classified as clinical markers of high value. Ki67 and tumor necrosis factor receptor 2 may become submarkers for disease prognosis in patients with PCa, its aggressiveness, and, consequently, attempts to determine a therapeutic strategy.

## 1. Introduction

One of the most frequently mentioned (second most common) causes of cancer-related mortality in men is prostate cancer (PCa) [1]. In 2022, more than 1.46 million estimated cases and approximately 396,000 deaths due to this cancer were recorded [2]. The incidence of prostate cancer varies significantly geographically. The highest age-standardized incidence rates (ASIRs) were noted in Northern Europe, Latin America and the Caribbean, North America, and Oceania. In contrast, the lowest rates were found in Africa and Asia. Mortality rates from prostate cancer also vary by region. The highest standardized mortality rates (ASMRs) were reported in Africa, followed by Latin America and the Caribbean, Europe, Oceania, North America, and Asia [3]. Analysis of data from 2000 to 2019 found that prostate cancer incidence rates were increasing in most countries included in this study (increasing in 65 countries, stable in 15, and decreasing in 9). Countries with a high human development index (HDI) have higher incidence rates, which may be related to better access to diagnostics. At the same time, lower mortality rates in these countries may be due to better access to treatment and healthcare [3].

Nearly 95% of prostate cancer cases originate in the epithelial tissue of the prostate (adenocarcinomas). It is a highly heterogeneous tumor with varying degrees of malignancy, and its architectural pattern is described as a Gleason score, which is used for prognostic evaluation [4].

Patients with PCa are treated differently depending on the patient’s general condition, clinical stage, and peripheral serum prostate-specific antigen (PSA) level. The first tests in patients with suspected prostate cancer are rectal examination and PSA determination as an organ-specific marker. PSA has differing values depending on the stage of the cancer, as well as during follow-up after treatment. This marker is variable in prostate diseases such as benign prostatic hyperplasia (BPH) and prostatitis. It is estimated that 25% of men with PCa do not have elevated serum PSA levels [5]. Clinicians have searched for and used various numerical models of PSA to determine clinical events and also to improve the clinical utility of the marker (PSA velocity, PSA density, PSA doubling) [6].

The treatment methods include radiotherapy (RT), surgery, hormonotherapy, and combinations of these methods. However, the method of treatment is determined by the division into the so-called risk groups. In the low-risk group, i.e., clinical advancement T1c-T2a, Gleason < 6, and PSA < 10 ng/mL, according to the National Comprehensive Cancer Network (NCCN) recommendations, active surveillance is considered an alternative to radical treatment—surgery or radiotherapy to the prostate gland only. In the intermediate risk group, i.e., clinical stage T2b-T2c, Gleason 7, and PSA 10–20 ng/mL, radiotherapy to the prostate area with half of the seminal vesicles and a short period of approximately 6 months of hormone therapy (HTH) using a luteinizing hormone-releasing hormone analog are recommended. However, in the high-risk group, i.e., clinical stage T3a-T4a, Gleason > 7, and PSA > 20 ng/mL, radiotherapy to the area of the prostate gland with seminal vesicles and the area of regional lymph nodes, as well as HTH for 18–36 months, is recommended. When interpreting tumor differentiation on the Gleason scale, the specifications of this scale should be taken into account, i.e., that a differentiation of 3 + 4 is prognostically better than 4 + 3. In doubtful situations, the presence of other unfavorable factors is taken into account (a tertiary component of Gleason differentiation %, perineural infiltration, cribriform structures, and the percentage of cancer-affected cores in the biopsy). Assuming the division into the so-called intermediate favorable (no more than one of the characteristics of the intermediate risk group and <50% of affected biopsies and Gleason score 3 + 4) and intermediate unfavorable risk, recommendations can be modified in subgroups. For example, in the indirectly beneficial group, HTH may be discontinued if the risk of adverse effects on the cardiovascular system increases. However, in the intermediate unfavorable group, elective radiotherapy of the lymphatic system may be considered. Different radical treatment methods are also associated with more or less manifest side effects, e.g., urinary incontinence; erectile dysfunction; and toxicity in the intestines, rectum, or bladder after radiotherapy. Hence, the careful and appropriate selection of treatment is more important and, therefore, the search for an effective biomarker that can predict the prognosis of disease is still ongoing [7].

Currently, it is a priority to develop prognostic biomarkers that would allow the identification of patients with slowly progressing prostate diseases with a low risk of progression, so that unnecessary treatment and patient burden can be avoided. In the future, such indicators could provide information about response and resistance factors in therapy. More and more attention is being paid to understanding the dynamics of changes and expression of many different proteins in the development of prostate cancer and the process of angiogenesis. The analyzed markers of inflammation are also indicators of cellular processes; however, they are currently in the research panel. Inflammatory markers such as C-reactive protein (CRP), tumor necrosis factor (TNF) receptors, or p-selectin are dynamic information in the patient’s blood serum, the concentration of which may depend on the advancement of the disease [8].

The Ki-67 protein is well known and widely utilized as a proliferation marker of tumor cells [9]. Some studies reported Ki-67 as a prognostic factor of survival and biochemical recurrence of prostate cancer [10,11,12]. In breast cancer, Ki-67 directly influences the choice of treatment method. The mitotic index in steroid-dependent cancers (having estrogen and progesterone receptors) differentiates biological subtypes into Luminal A and Luminal B, which determines the choice of systemic treatment method in these patients. In breast cancer, the dynamics of Ki-67 changes (so-called DKi67%) are assessed during neoadjuvant therapy, which is a strong predictor of complete pathological response and a prognostic factor also for overall survival [13].

Similar correlations should be used in the course of prostate cancer and treatment selection. Such information would be particularly important for patients who require hormone therapy and struggle with cardiovascular diseases.

This prospective study was intended to assess the clinical features and concentrations of selected mediators of apoptosis and inflammation, and immunoexpression of Ki67 and selected mediators of inflammation in patients with PCa after prostatectomy procedures, patients who underwent palliative radiotherapy for bone metastases, and patients with BPH.

## 2. Materials and Methods

### 2.1. Patients

This study was conducted in the Department of Urology and the Department of Radiotherapy at the Multispeciality Hospital in Gorzow (Poland) in 2019. A total of 88 patients, including 54 patients with PCa and 34 healthy volunteers with benign prostatic hyperplasia (BPH), were recruited for this study. All 54 patients had histopathologically confirmed and clinically diagnosed PCa, which was then categorized according to clinical stage. Prior to treatment, all patients had a histopathological result from a biopsy.

Of the patients with PCa, 35 underwent prostatectomy (PCa surgical group) and 19 received palliative radiotherapy for bone metastases (PCa palliative group). None of the patients had any other type of tumor. In a group of 34 volunteers (BPH group), BPH was histologically confirmed. Patients with neoplasms and enlarged lymph nodes for any reason were excluded.

Patients in the surgical group were not treated with chemotherapy or endocrine therapy before surgery. Diseases that would prevent surgery were also excluded. Patients who qualified for laparoscopic prostatectomy had previously undergone full diagnostics. The disease was limited to the organ without enlarged lymph nodes. This group of patients was prepared for laparoscopic prostatectomy and, therefore, before the surgery, a full medical history was conducted with the patient, then a physical examination with a per rectum examination, laboratory tests and imaging tests such as magnetic resonance imaging of the small pelvis to assess the prostate gland, seminal vesicles, bladder, rectum and lymph node status. A prostate biopsy was performed to confirm the cancer. During the laparoscopic procedure, the entire prostate gland, seminal vesicles, part of the urethra, and tissues with pelvic lymph nodes were removed, limiting the interference in the patient’s body to a minimum. This method is minimally invasive and is therefore performed only in patients with a low stage of advancement, i.e., disease limited only to the prostate gland. All collected postoperative material, including the prostate gland, seminal vesicles, and tissue with lymph nodes, was evaluated histopathologically. In the surgical group, lymph nodes and surgical margins were not affected by cancer (N0, M0, and R0).

Palliative patients received endocrine therapy as well as three-dimensional conformal radiotherapy with conventional hypofractionation in the range of 4–8 Gy, giving a total dose of 6–20 Gy, for bone metastases. The procedure was performed using a 6 MV photon beam. Patients included in this group did not have visceral metastases.

**Figure 1:**
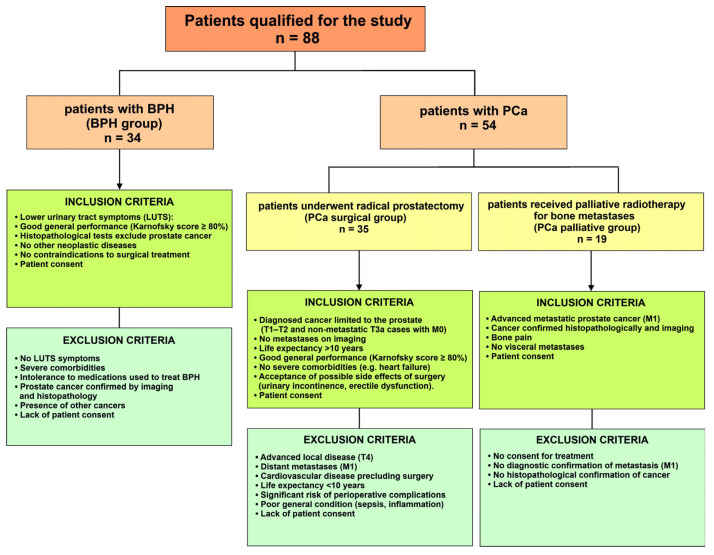
Patient inclusion and exclusion selection criteria in individual groups. BPH—benign prostate hyperplasia; PCa—prostate cancer; LUTS—lower urinary tract symptoms; T—in the TNM system, parameter specifically describing the size and extent of the primary tumor; M—in the TNM system, parameter refers to whether the cancer has metastasized.

Patient inclusion and exclusion selection criteria in individual groups included the patient’s clinical and functional parameters (Figure 1).

Data on clinical stage, Gleason score, PSA level, and scan-based prostate volume were collected for each patient. Peripheral serum samples were collected before and after surgery, as well as before the start of radiotherapy. To evaluate clinical factors, blood tests (PSA), biopsies, and radiological evaluation such as magnetic resonance imaging (MRI) pelvis were used, and gallium-68 (Ga-68) prostate-specific membrane antigen (PSMA) positron emission tomography/computed tomography (PSMA PET/CT).

This study was approved by the Ethics Committee of the District Medical Council in Zielona Góra, No. 18/129/2019. Written informed consent was obtained from all subjects involved in this study (patients and healthy volunteers). Written consent was provided by patients at the time of tissue collection to the clinical teams.

### 2.2. Blood Collection

Blood samples were collected from patients in the morning (between 7:00 and 8:00 a.m.) from the middle basilic vein by use of S-Monovette tubes (Sarstedt AG & Co., Nümbrecht, Germany). Within 60 min of collection, the material was centrifuged at +4 °C at 1000× *g* for 20 min. The obtained serum sample fractions were then aliquoted and frozen at −80 °C until further laboratory analysis.

### 2.3. Analysis of Apoptotic and Inflammatory Mediators

An enzyme-linked immunosorbent assay (ELISA) was used to determine apoptosis and inflammatory mediators in the serum of patients. Tumor necrosis factor α (TNFα), high-sensitivity C-reactive protein (hs-CRP), TNF receptor type 1 (TNFRI) and type 2 (TNFRII), caspase 8 (Cas8), caspase 9 (Cas9), Fas ligand (FasL) and TNF-related apoptosis-inducing ligand (TRAIL), P-selectin, total circulating cell-free DNA (cfDNA) concentrations, and DNA methylation (metDNA) in serum were measured according to the manufacturer’s instructions. Detection limits and other parameters were previously described in detail by Wolny-Rokicka et al. [8].

### 2.4. Histological Analysis

Obtained samples of prostates were routinely fixed in 4% buffered paraformaldehyde and embedded in paraffin blocks. Next, 3 μm thin sections were cut and placed on the polylysine-coated slides for further analysis.

### 2.5. Immunohistochemistry

Prostate tissue sections were first deparaffinized and rehydrated, followed by antigen retrieval by boiling the samples in Target Retrieval Solution (Dako, Glostrup, Denmark) at pH 9.0 for 30 min. To block endogenous peroxidase activity, Peroxidase Blocking Solution (Dako) was applied. The sections were then incubated with primary antibodies: mouse monoclonal anti-human Ki67 (clone MIB-1; Dako, Glostrup, Denmark) at a 1:100 dilution, rabbit polyclonal anti-TNFRI (Abcam, Cambridge, UK) at 1:20, and rabbit polyclonal anti-TNFRII (Abcam, Cambridge, UK) also at 1:20. Incubation with primary antibodies was carried out for 30 min in a humid chamber. This was followed by incubation with a secondary antibody conjugated to horseradish peroxidase (Dako, Glostrup, Denmark). Immunoreactivity was visualized using diaminobenzidine. Finally, the sections were counterstained with Mayer’s hematoxylin (Sigma-Aldrich, St. Louis, MO, USA), dehydrated, and mounted with coverslips. Immunohistochemical evaluation was performed under a light microscope (Olympus BX 41, Hamburg, Germany).

### 2.6. TUNEL Assay

Apoptosis was assessed using the TUNEL (Terminal deoxynucleotidyl transferase-mediated dUTP nick end labeling) method with the In Situ Apoptosis Detection Kit (Merck Millipore, Billerica, MA, USA). Tissue sections were first deparaffinized and rehydrated, followed by treatment with proteinase K (Dako, Glostrup, Denmark) to expose antigenic sites. Endogenous peroxidase activity was blocked by incubating the sections for 10 min in a humid chamber with peroxidase blocking solution (Dako, Glostrup, Denmark). Subsequently, the sections underwent sequential incubation: first for 60 min with terminal deoxynucleotidyl transferase, and then for 30 min with anti-digoxigenin antibodies, both steps performed in a humid chamber. The reaction was visualized using 3,3′-diaminobenzidine (Dako, Glostrup, Denmark), and the sections were counterstained with Mayer’s hematoxylin. Between each stage, the sections were rinsed in phosphate-buffered saline (PBS). Finally, the slides were dehydrated and coverslipped.

### 2.7. Computer Analysis of Immunohistochemistry

Prostate sections were scanned with a ScanScope AT2 scanner (Leica Microsystems, Wetzlar, Germany) at 400× magnification, achieving a resolution of 0.25 μm/pixel. The resulting digital images of the slides were visually analyzed on a computer screen using ImageScope Viewer software(version 11.2.0.780) (Aperio Technologies, Vista, CA, USA). Quantitative analysis of Ki67 immunoexpression was performed using the nuclear v9 algorithm with settings calibrated to match visual assessment, with brown nuclear staining considered a positive result. For the analysis of TNFα, TNFRI, and TNFRII expression, a cytoplasmic v2 algorithm (Aperio Technologies, Vista, CA, USA) was used. The images of prostate sections with brown or orange, and yellow pixels indicated positive immunostaining for cell cytoplasm (strong, moderate, and weak intensity, respectively). Using an algorithm, the percentage of cells with weak, moderate, and strong positive immunostaining in the cytoplasm was automatically determined. Analysis areas were manually defined. The percentage of cells with the expression of Ki67, TNFRI, and TNFRII was counted in each patient (a total of 88 random fields: 35 in the PCa surgical group, 19 in the PCa palliative group, and 34 in the BPH group).

### 2.8. Statistical Analysis

The obtained variables were analyzed using Statistica 13.0 software (TIBCO Software, Palo Alto, CA, USA) for each of the evaluated parameters. The means, standard deviations, medians, and ranges were calculated. The percentages of Ki67- and TUNEL-positive cells, and cells with weak, moderate, and strong immunoexpression of TNFα, TNFRI, and TNFRII are presented in bar charts as medians and ranges.

The values were analyzed for normality using the Shapiro–Wilk test. Data for age and prostate volume met the assumptions of normal distribution and equal variances (Brown-Forsyth tests); therefore, the F test (one-way analysis of variance) and Tukey’s post hoc test were used.

The obtained values for PSA; Ki67- and TUNEL-positive cells; CRP; P-selectins; TNFα; TNFRI; TNFRII; Fas L; TRAIL; Caspase 8; Caspase 9; cfDNA; met-DNA; and the percentage of cells with weak, moderate, and strong immunoexpression of TNFα, TNFRI, and TNFRII failed the normal distribution assumption, and the non-parametric Kruskal–Wallis test followed by Dunn’s multiple comparison tests for post hoc analysis were used to evaluate the differences between the groups. Differences at *p* < 0.05 were considered to be statistically significant.

Spearman’s rank correlations were used to assess the relationship between variables. A Spearman’s rank correlation coefficient (r_s_) value ranging from 0.10 to 0.29 was defined as weak, from 0.30 to 0.49 as average, from 0.50 to 0.69 as high, and from 0.70 to 1.00 as very high.

Receiver operating characteristic (ROC) curves were plotted as sensitivity vs. specificity for Ki67. The area under the curve (AUC) was used to overall separation of patients with poor prognosis from those eligible for surgery. Youden’s index was assessed to obtain the optimum cut-off point for the Ki67 proliferation index.

## 3. Results

### 3.1. Baseline Characteristics of the Patients

There were no statistically significant differences in the age of PCa surgical and PCa palliative patients with prostate cancer in comparison to the BPH patients. In PCa surgical and PCa palliative patients, PSA concentration was significantly higher (*p* < 0.001) vs. the BPH group. Both in PCa surgical and PCa palliative patients, the prostate volume was significantly lower (*p* < 0.001, *p* = 0.003, respectively) vs. the BPH group (Table 1).

In the PCa surgical patients, men with Gleason scores 1–6 predominate (42.9%), whereas in the PCa palliative group of patients, men with Gleason scores 8–10 predominate (47.4%). In the PCa surgical group, most patients have stage T2b (34.3%), and a significant percentage have stage T1c and T2a (20.0% each). In the PCa palliative group, stage T2c predominates (57.8%). Additionally, in the palliative group, patients with T3 and T4 stages were noted (Table 2).

### 3.2. Analysis of Proliferation and Apoptotic Index

**Figure 2:**
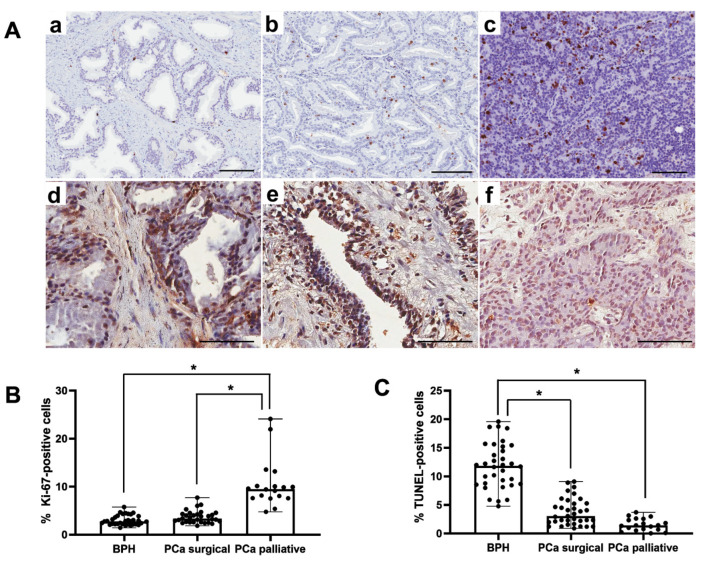
Representative light micrographs of Ki67 immunoexpression (**a**–**c**) and reaction showing TUNEL-positive cells (**d**–**f**), both characterized by brown-stained nuclei, in the prostate from patients in the BPH group (**a**,**d**), in the PCa surgical group (**b**,**e**) and the PCa palliative group (**c**,**f**). Scale bar—100 μm (**A**). Comparison of percentage of Ki67-positive (**B)** and TUNEL-positive (**C**) cells in the patients with BPH and prostate cancer after prostatectomy procedures and palliative radiotherapy. BPH—patients with benign prostate hyperplasia; PCa surgical—operated patients with adenocarcinoma; PCa palliative—patients with adenocarcinoma not eligible for surgery, who received radiotherapy for bone metastases; *—*p* < 0.001 (Kruskal–Wallis test).

In the BPH, PCa surgical, and PCa palliative patients, in all examined prostate glands, both Ki67-positive and TUNEL-positive cells were characterized by brown-stained cell nuclei (Figure 2A). In PCa palliative patients, the percentage of Ki67-positive cells was significantly higher (*p* < 0.001) vs. the BPH and PCa surgical group. In the PCa surgical patients, there were no statistically significant differences in the percentage of Ki67-positive cells vs. the BPH group (Figure 2B). Both in PCa surgical and PCa palliative patients, the percentage of TUNEL-positive cells was significantly lower (*p* < 0.001) vs. the BPH group. There were no statistically significant differences between PCa surgical and PCa palliative patients (Figure 2C).

In the PCa surgical and palliative patient groups, a statistically significant strong positive correlation was shown between proliferation index and Gleason score (Table 3). With the increase in the proliferative index, an increase in the Gleason score was observed (Table 4).

**Figure 3:**
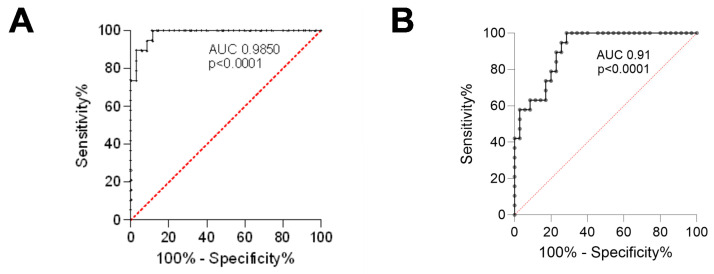
The ROC curves for the Ki67 proliferation index. (**A**) Separates the PCa palliative group from the PCa surgical group; (**B**) Separates patients with the Gleason score up to 7 from those above 7. ROC—receiver operating characteristic; AUC—area under the curve; PCa surgical—patients operated on with adenocarcinoma; PCa palliative—patients with adenocarcinoma not eligible for surgery, who received radiotherapy for bone metastases.

The optimal cutoff point for the Ki67 proliferation index, which best separates patients with poor prognosis (PCa palliative group) from those eligible for surgery (PCa surgical group), is approximately 4.74% (Figure 3A). In turn, the optimal cutoff point for the Ki67 proliferation index, which best separates patients with the Gleason score up to 7 from above 7, is approximately 3.91% (Figure 3B).

### 3.3. Markers of Inflammation

In PCa surgical and PCa palliative patients, the concentration of CRP was significantly higher (*p* < 0.001 and *p* = 0.027, respectively) vs. the BPH. In the case of P-selectin concentration, statistically significant differences (*p* = 0.019) only in the PCa palliative patients vs. BPH were noted. In the PCa surgical patients, the concentration of TNFRII was significantly lower vs. the BPH and PCa palliative group (*p* = 0.004 and *p* < 0.001, respectively). In the case of TNFα, TNFRI, and Fas L, there were no statistically significant differences between the groups (Table 5).

**Figure 4:**
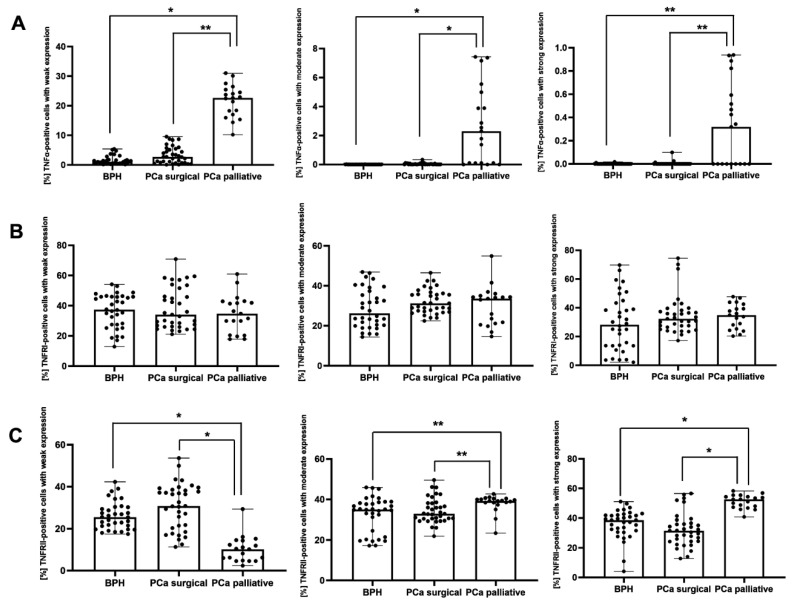
The percentage of cells with weak, moderate, and strong immunoexpression of TNFα (**A**), TNFRI (**B**), and TNFRII (**C**) in the patients with BPH and prostate cancer after prostatectomy procedures and palliative radiotherapy. BPH—patients with benign prostate hyperplasia; PCa surgical—operated patients with adenocarcinoma; PCa palliative—patients with adenocarcinoma not eligible for surgery, who received radiotherapy for bone metastases; TNFα—tumor necrosis factor α; TNFRI—tumor necrosis factor receptor 1; TNFRII—tumor necrosis factor receptor 2; *—*p* < 0.001; **—*p* < 0.05 (Kruskal–Wallis test).

Both in BPH and PCa surgical patients, the percentage of TNFα-positive cells with weak and moderate immunoexpression was significantly lower (*p* < 0.001, *p* = 0.015, respectively) vs. the PCa palliative group. In the PCa palliative group, the percentage of TNFα-positive cells with strong immunoexpression was significantly higher vs. PCa surgical and BPH patients (*p* = 0.011 and *p* = 0.007, respectively) (Figure 4A). There were no significant differences in the percentage of TNFRI-positive cells with weak, moderate, and strong immunoexpression between the groups (Figure 4B).

Both in BPH and PCa surgical patients, the percentage of TNFRII-positive cells with weak immunoexpression was significantly lower (*p* < 0.001), and TNFRII-positive cells with moderate immunoexpression were significantly higher (*p* = 0.020, *p* = 0.021, respectively) vs. the PCa palliative group. In the PCa palliative group, the percentage of TNFRII-positive cells with strong immunoexpression was significantly higher (*p* < 0.001) vs. BPH and PCa surgery patients (Figure 4C).

### 3.4. Markers of Apoptosis

In PCa surgical patients, the concentration of Caspase 8 was significantly lower (*p* = 0.045) vs. the BPH group. In the case of concentration of met-DNA, statistically significant differences (*p* = 0.027) were observed only in the PCa palliative patients vs. BPH. In the case of TRAIL, Caspase 9, and cfDNA, there were no statistically significant differences between the groups (Table 6).

### 3.5. Correlations Between Selected Parameters

In the BPH group of patients, a statistically significant positive correlation was only shown between prostate volume and TNFa concentration (Table 7).

## 4. Discussion

Our study is unique because we compare both pro-inflammatory factors and factors responsible for the assessment of proliferation and apoptosis in prostate cancer cells in patients who underwent various paths of clinical management due to the advancement of the disease. In this study, we investigated whether or not it had a direct effect on the extremely advanced group compared to the locally advanced group or the group without confirmed cancer, and their correlation.

An attempt was made to effectively explain the prognosis of patients with prostate adenocarcinoma based on the Ki67 index and the Gleason score. The Ki-67 antigen is exclusively reactive for the nuclear structure only in the proliferating cells, not in resting cells in the G0 phase of mitosis [14] and, therefore, is utilized in diagnosing malignancies in the breast, lung, brain, and stomach [15,16,17]. Moreover, these studies demonstrated that the Ki-67 index can be utilized as a valuable prognostic biomarker.

In our study, Ki-67 expression was associated with the palliative PCa group and was significantly increased compared with the BPH group and the surgical PCa group. This finding is in agreement with Verma et al. [18] and Rashed et al. [19], who also found that this marker is highly expressed in prostate cancer as compared with BPH. In another study [12], it was observed that the expression of Ki-67 was significantly associated with postoperative adverse pathologic outcomes in the surgical patients. Furthermore, a higher Ki-67 proliferation index was found to strongly and positively correlate with the Gleason score in the surgical and palliative PCa groups. The prognostic intervals of Ki-67 in Gleason grades of low, intermediate, and high risk reflect the representativeness of Ki-67 results in both palliative and surgical PCa groups. Consistent with previous data in the literature [11,20], Ki-67 provided prognostic information and showed a very strong association with distant metastases, cause-specific death, and all-cause death in prostate cancer patients. It has even been suggested that Ki67 > 7.1% is associated with a high risk of distant metastasis.

Ki-67 as a marker of tumor cell proliferation activity can be used as an additional indicator of tumor aggressiveness and thus may influence the choice of treatment strategy and patient prognosis. This marker, when defining the range of percentage cut-off points, would correlate with the assessment of the stage of advancement and treatment efficacy. In the study, Mesko et al. [21] found that Ki-67 significantly differed between risk groups and Gleason points (mean Ki-67 for Gleason points 6, 7, and ≥8: 5.0% ± 3.8%, 7.7% ± 7.0% and 12.0% ± 12.4%). Our study showed the % Ki-67 range increased with the Gleason score in both the PCa surgical and palliative groups. An attempt was also made to determine the Ki-67 value range for Gleason 7 and >7. In the group of patients undergoing surgery and in the palliative group for Gleason scores up to 7 and >7, the Ki-67 cut-off point was 3.91%. In turn, a specific cut-off value for Ki-67 established in the context of prognosis (surgical or palliative therapy) was 4.74%. Assessment based on classical parameters—Ki67 index and Gleason scale—used together suggests that increased Ki67 and a Gleason score above 7 indicate greater aggressiveness, which requires more intensive monitoring and adjuvant therapy, such as radiotherapy or hormone therapy. This allows for the future identification of patients at increased risk of metastasis or recurrence.

This study showed a significant relationship between high Ki-67 expression and a worse prognosis group (cancer-specific and overall survival) in patients with higher Ki-67 expression. Similarly, in the group of patients with prostate cancer, it was found that the high Ki-67 expression was significantly related to early progression and disease recurrence, distant metastases, and disease-specific survival [20,22].

In recent years, research on complex molecular profiles has been developed, e.g., RNA profile studies have shown that differences in the expression of some genes together with a high Ki67 index can more accurately predict tumor aggressiveness, which allows for more precise therapy adjustment [23].

Disorders in the balance between proliferation and apoptosis of prostate cells lead to the constant growth of stromal and epithelial elements, which is one of the reasons for the development of BPH [24]. The consequence of disturbances between these processes will be far-reaching neoplastic changes. And that is why the TUNEL method was used to assess the phenomenon of apoptosis to quantify it in individual groups of patients. The method of terminal deoxynucleotidyl transferase (TdT) dUTP Nick-End Labeling (TUNEL)—described in 1992 [25]—consists of the template-independent identification of blunt ends of double-stranded DNA breaks by TdT. The enzyme catalyzes the addition of labeled dUTPs to 3 c-hydroxyl termini of DNA ends and can be visualized using immunohistochemical techniques. This method has been designed to detect apoptotic cells that undergo extensive DNA degradation during the late stages of apoptosis [26]. In our study, the number of TUNEL-positive cells decreased in the group where the Ki67 parameter increased. Both in PCa surgical and PCa palliative patients, the percentage of TUNEL-positive cells was significantly lower vs. the BPH group (*p* < 0.001). Uncontrolled cancer growth may also be caused by a reduction in programmed cell death, or apoptosis [27]. It happens, however, that these processes get out of control and there are uncontrolled reactions leading to neoplastic growth, which is manifested by an increase in subsequent markers, such as an increase in the PSA antigen. In our study, PSA antigen, as expected, had higher concentrations in blood serum in patients with prostate cancer before surgery and in the PCa palliative group.

Prostate volume was significantly lower in prostate cancer patients (PCa surgical and PCa palliative group) vs. the control groups. In BPH, there is a generalized, diffuse enlargement of the prostate, whereas prostate cancer often develops in a more localized manner (often in the peripheral zone of the gland), which may result in a smaller overall volume of the gland. Our study showed that larger prostate volumes in patients with BPH are positively correlated with TNF-alpha, which may result from increased inflammatory and proliferative processes in the glandular tissue.

Inflammatory processes are the first indicators that manifest the environmental changes occurring in the cell. The determination of such indicators that would be important and helpful in clinical management is tempting due to the easy availability of such a test in the form of a liquid biopsy. It is also important to correlate such indicators with other markers to detect the initiating changes of a neoplastic nature earlier. Immune cell response to inflammation promotes the development and progression of several types of solid cancer, including prostate cancer [28,29]. Inflammatory infiltration favors a preneoplastic environment. Various bioactive molecules are delivered there, such as epidermal growth factor, transforming growth factor-β, tumor necrosis factor, fibroblast growth factors, interleukins, chemokines, histamine, and heparins [30]. There are changes in the functioning of immune cells, which may contribute to the induction of pro-cancer mutations and the induction of resistance to apoptosis [31]. In the study, de Bono et al. observed recruitment of leukocytes (macrophages, lymphocytes, granulocytes, and monocytes) to the prostate in the prostate cancer-driven inflammation responses [32]. The inflammation markers such as CRP, p-selectin, and TNFRII were revealed to be independently significant prognostic markers in this study. In PCa surgical and PCa palliative patients, CRP concentration was significantly higher than in the BPH group. Tumor necrosis factor is widely accepted as a tumor-suppressive cytokine via its ubiquitous TNFRI. TNFRI and TNFRII possess different functions when bound to TNFα due to differences in their intracellular structures, such as TNFRII lacking a death domain (DD) [33]. The TNFRII receptor is not only expressed in some tumor cells but also suppressive immune cells (e.g., regulatory T cells and myeloid-derived suppressor cells) [34,35,36]. The TNFRII receptor (in contrast to TNFRI) diverts the tumor-inhibiting TNF into a tumor. TNFRII directly promotes the proliferation of some kinds of tumor cells. This receptor activates immunosuppressive cells, which enables the development of cancer [37]. In the conducted study, an analogy to the above-mentioned studies can be found. In surgery patients, the TNFRII concentration was significantly lower vs. the BPH and PCa palliative group. In the PCa palliative group, an upward trend was observed for TNFα compared to the BPH and PCa surgical groups. There were no statistically significant differences in the TNFR1 and Fas L concentration in the BPH, PCa surgical, and PCa palliative groups. P-selectin is a type of cell adhesion molecule that plays a crucial role in the inflammatory response and the process of blood clotting. It is localized in platelet granules and Weibel–Palade bodies of vascular endothelium. P-selectin helps in the initial inflammation and is induced by inflammatory mediators. Its main task is to bind circulating leukocytes to vascular endothelium during the inflammatory response to sites of infection or injury. It also plays a role in the formation of blood clots [38]. Several studies [39,40] have reported P-selectin expression as an increased marker on the platelet surface in such cancer types as breast, lung, kidney, and colon cancers. Other studies have reported opposite conclusions. They found no difference in this marker in the serum of prostate cancer patients compared to BPH controls [41] or showed an increase in P-selectin in the palliative group and a decrease in the group with locally advanced cancer undergoing surgery [8]. Our current study showed that the P-selectin level decreased in the PCa palliative group vs. the BPH group, while in PCa surgical patients, statistically insignificant differences were noted. P-selectin and its role as a marker are ambiguous for clinical use.

This study also tried to evaluate some indicators of the apoptosis process. Among the apoptosis markers (listed in Table 4), only met-DNA and Caspase 8 were significant in the study. Caspase 8 is an important enzyme in programmed cell death, or apoptosis. Our study showed statistically significantly higher Caspase 8 values in the BPH group compared to the PCa surgical patient group. This result significantly indicates increased apoptosis processes in the non-cancer group, because these processes are disturbed in cancers [42]. The apoptosis program of cancer cells is stabilized by proteasome inhibitors, which leads to the stabilization of caspase 8 [43]. The effect of inhibitory proteasomes is used in pharmacology to induce apoptosis [44,45]. In a study by Thorpe et al. [46] on several prostate cancer cell models and functional prostate epithelial cells, proteasome inhibition sensitized prostate cancer cells to apoptosis.

DNA methylation is part of the phenomenon of epigenetics. Abnormal DNA methylation is associated with cancer development through silencing of tumor suppressor genes or activation of oncogenes [47,48]. Hypermethylation at gene loci in PCa tissues compared to BPH is involved in cancer progression [49]. In our study, opposite to another study, statistically significantly higher met-DNA values were obtained in the group of patients with BPH compared to the PCa palliative group, without statistically significant differences in the PCa surgical group.

The latest developments in the possibilities of discovering new molecular biomarkers include using genomics, transcriptomics, and artificial intelligence [48]. As presented, there is clinical evidence suggesting the importance of types of molecular markers—coding and non-coding genes. But it is important to establish the types of mutations that can be affected. It is expected to identify new biomarkers for differentiation of indolent and aggressive disease, for prognosis and prediction to improve clinical treatment of patients [50]. Other studies [51] are looking for a relationship between obesity and inflammatory infiltrates of the prostate, which may be a consequence of autophagy deregulation in obese patients. Hence, in the prophylaxis of cancer disease, physical activity is important. Interestingly, adipokines measured in men with metastatic castration-resistant PCa treated with androgen deprivation therapy with the addition of androgen signaling inhibitors (ASI) showed a positive correlation with the amount of host adipose tissue and the efficacy of treatment and the toxicity of ASI [52]. Thus, markers are sought that could be used for rapid and effective assessment of patients and that would become a permanent algorithm for clinical management.

This study has limitations, including the relatively small number of patients. One of the reasons is the use of certain inclusion and exclusion criteria applied in order to minimize potential confounding factors and to obtain a homogeneous study group. We focused on patients meeting clearly defined clinical and histopathological criteria, excluding incomplete clinical data. Such an approach, although it limited the size of the study population, was necessary to ensure that the observed relationships were not biased by heterogeneous patient characteristics. We believe that this strategy strengthens the internal validity of our findings, and it provides a solid basis for future studies involving larger and more diverse patient cohorts. The absence of data on patient survival prevented us from performing survival analyses, which would have provided valuable information on the prognostic significance of the studied parameters. Nevertheless, we recognize that survival outcomes are a key aspect in this type of research, and we intend to include such analyses in future studies involving larger, well-characterized patient cohorts and longer follow-up periods.

## 5. Clinical Implications

Interestingly, many patients do not need radical treatment because there are patients who will die “with” and not “because of” PCa, and, therefore, a significant proportion of patients will receive radical treatment without the need [53]. It has been shown that about 40–60% of patients with high-risk features will not be successful in the treatment [54]. In this context, there is a clear need that PCa research should focus on implementing biological markers (e.g., markers of apoptosis, proliferation, and inflammation) into existing clinical procedures, which may involve addressing prognostic and therapeutic benefits. The compiled evaluation of tumor proliferation and apoptosis, and markers of inflammation, may constitute an approach that will allow for a better determination of PCa dynamics and the use of broader diagnostics. The assessment of markers of inflammation may create opportunities for incorporating targeted therapies aimed at modulating the tumor microenvironment. Monitoring of the mentioned markers may support an individual approach to patient diagnostics and making appropriate decisions regarding systemic therapy (e.g., adding immunotherapy or radiotherapy). Implementation of the studied markers into standard clinical practice might allow the development of personalized treatment plans while optimizing therapeutic efficacy and minimizing adverse effects. Moreover, it may enable not only earlier detection of tumor progression but also provide additional clinical data on treatment toxicity and its effectiveness.

## 6. Future Directions

In patients with PCa, in addition to making decisions based solely on parameters such as PSA and Gleason score, molecular biomarker panels should be considered. The use of these markers in the clinical evaluation of patients before treatment can enable the selection of therapy with optimized toxicity, especially in patients with a high risk of complications. Moreover, in view of the increasing risk factors for prostate cancer, there is a need to implement screening tests for this disease in men, as well as preventive programs. Prospective studies are also needed that integrate biological data with genomic, epigenomic, and transcriptomic biomarkers and artificial intelligence technologies that will be able to assess the risk of PCa, predict treatment response, classify patients into individual treatment strategies (radical or palliative), and generate personalized survival prognoses.

## 7. Conclusions

The performed study suggested that Ki-67 and TNFRII expression, and the number of apoptotic cells with DNA fragmentation may constitute significant biological differentiating markers. Ki-67 expression correlated positively with the Gleason score and was clearly higher in patients undergoing palliative treatment, while showing an inverse relationship with apoptotic activity assessed by the TUNEL method. These markers may have clinical value as independent indicators of the biological aggressiveness of the tumor. However, in order to confirm the obtained relationships, further studies should be carried out on a larger number of patients, taking into account potential confounding factors, differences in demographic and clinical characteristics.

In light of these observations, it seems reasonable to routinely measure Ki-67 already at the stage of initial diagnostics—in parallel with PSA assessment and histopathological examination—as an auxiliary factor in qualifying for more intensive oncological treatment. This may facilitate decision-making on the implementation or abandonment of therapies with a higher potential for toxicity, such as high-dose radiotherapy or long-term hormonal treatment.

In turn, TNFRII showed the highest level of expression in the inflammatory microenvironment of the metastatic tumor. Since changes detected in blood serum reflect ongoing processes within the tissues, the expression of inflammatory molecules—including TNFRII—may constitute early indicators of disease progression. Their profile in the systemic environment may constitute so-called submarkers, useful for prognosticating the course of the disease, assessing its aggressiveness, and making therapeutic decisions.

The Gleason score defines oncological risk in prostate cancer patients, dividing cases as low, favorable intermediate, unfavorable intermediate, and high risk. Our study revealed an association between Ki-67 expression with the degree of malignancy of the tumor. We established specific cut-off values for Ki-67 in the context of prognosis (surgical or palliative therapy) and the Gleason score. In the mentioned cases, the Ki-67 cut-off points were found 4.74% and 3.91%, respectively, which may have clinical importance, especially in situations where the Gleason score is not precisely described or/and double control of malignancy tumor was established (in consequence there is a need for additional verification of the histological grade of the tumor). This approach might allow for more precise assessment of the risk and more effective adjustment of the therapeutic strategy for the patient.

## Figures and Tables

**Table 1:** Comparison of age, PSA concentration, and prostate volume in the patients with BPH and prostate cancer after prostatectomy procedures and palliative radiotherapy.

Parameter	BPH (*n* = 34) Median (Range) X ± SD	PCa Surgical (*n* = 35) Median (Range) X ± SD	PCa Palliative (*n* = 19) Median (Range) X ± SD
Age (years)	70 (51–82) 68.2 ± 7.6	67 (53–82) 67.1 ± 5.9	68 (55–88) 68.3 ± 8.2
PSA (ng/mL)	3.0 (0.2–14.0) 3.6 ± 2.9	8.0 (4.2–67.0) 11.1 ^a^ ± 11.7	24.0 (4.4–616.0) 93.0 ^a^ ± 146.0
P_vol_ (cm^3^)	64.0 (20.0–100.0) 63.9 ± 21.2	41.0 (17.0–80.0) 45.4 ^a^ ± 17.0	43.0 (20.0–84.0) 45.3 ^b^ ± 19.4

BPH—patients with benign prostate hyperplasia; PCa surgical group—operated patients with adenocarcinoma; PCa palliative—patients with adenocarcinoma not eligible for surgery, who received radiotherapy for bone metastases; PSA—prostate-specific antigen; Pvol—prostate volume; X ± SD − arithmetical mean ± standard deviation; ^a^—*p* ≤ 0.001 vs. BPH; ^b^—*p* < 0.05 vs. BPH (F test (age, P_vol_) and Kruskal–Wallis test (PSA), respectively).

**Table 2:** Comparison of Gleason score and pathological stage in the patients with prostate cancer after prostatectomy procedures and palliative radiotherapy.

Parameter	PCa Surgical Number of Patients (%)	PCa Palliative Number of Patients (%)
Gleason score		
1–6	15 (42.9%)	5 (26.3%)
7	14 (40.0%)	5 (26.3%)
8–10	6 (17.1%)	9 (47.4%)
Pathological stage		
T1a	0 (0.0%)	0 (0.0%)
T1b	0 (0.0%)	0 (0.0%)
T1c	7 (20.0%)	1 (5.3%)
T2a	7 (20.0%)	0 (0.0%)
T2b	12 (34.3%)	1 (5.3%)
T2c	6 (17.1%)	11 (57.8%)
T3a	3 (8.6%)	2 (10.5%)
T3b	0 (0%)	1 (5.3%)
T4	0 (0%)	3 (15.8%)
N0	35 (100.0%)	10 (52.6%)
N1	0 (0.0%)	9 (47.4%)
M0	35 (100.0%)	0 (0.0%)
M1a	0 (0.0%)	0 (0.0%)
M1b	0 (0.0%)	19 (100%)
M1c	0 (0.0%)	0 (0.0%)

PCa surgical—operated patients with adenocarcinoma; PCa palliative—patients with adenocarcinoma not eligible for surgery, who received radiotherapy for bone metastases.

**Table 3:** Correlation between proliferation index (percentage of Ki67-positive cells) and selected histopathological parameters in the patients with prostate cancer after prostatectomy procedures and palliative radiotherapy.

Parameter	PCa Surgical	PCa Palliative
Gleason score	r_s_ = 0.885 *p* < 0.001	r_s_ = 0.839 *p* < 0.001
Pathological stage	r_s_ = 0.086 *p* = 0.621	r_s_ = 0.203 *p* = 0.450

PCa surgical—operated patients with adenocarcinoma; PCa palliative—patients with adenocarcinoma not eligible for surgery, who received radiotherapy for bone metastases. r_s_—Spearman’s rank correlation coefficient, *p*—statistical significance (Spearman’s rank correlation).

**Table 4:** Range of percentage of Ki-67-positive cells in the patients with prostate cancer after prostatectomy procedures and palliative radiotherapy.

Range [% of Ki67-Positive Cells]			
Gleason score	≤6	7	>7
PCa palliative	4.77–7.53	7.60–9.86	9.14–24.12
PCa surgical and palliative	1.87–7.53	2.50–9.86	4.11–24.12

PCa surgical—operated patients with adenocarcinoma; PCa palliative—patients with adenocarcinoma not eligible for surgery, who received radiotherapy for bone metastases.

**Table 5:** Comparison of the concentration of selected inflammation markers detected by ELISA method in serum of the patients with BPH and prostate cancer after prostatectomy procedures and palliative radiotherapy.

Parameter	BPH Median (Range) X ± SD	PCa Surgical Median (Range) X ± SD	PCa Palliative Median (Range) X ± SD
CRP	3.4 (0.4–16.7) 4.6 ± 4.2	6.6 (1.6–12.9) 6.8 ^b^ ± 3.4	9.2 (2.0–13.7) 9.1 ^a^ ± 3.7
P-selectins	14.2 (0.9–22.9) 14.7 ± 3.9	13.7 (6.5–21.7) 13.9 ± 3.8	12.2 (7.4–18.9) 12.2 ^b^ ± 3.2
TNFα	235.6 (37.3–1247.9) 383.1 ± 363.5	229.3 (0.7–721.5) 266.6 ± 164.9	278.6 (145.3–1663.9) 557.5 ± 552.0
TNFRI	55.3 (49.3–514.3) 86.3 ± 114.8	51.3 (46.3–110.3) 57.0 ± 16.1	51.8 (48.3–59.3) 52.0 ± 3.1
TNFRII	1337.4 (1100.3–2101.7) 1368.4 ± 240.4	1157.4 (806.0–1368.9) 1128.0 ^b,c^ ± 150.1	1485.3 (1101.7–1957.4) 1507.3 ± 248.4
Fas L	0.11 (0.09–0.16) 0.12 ± 0.01	0.12 (0.11–0.29) 0.13 ± 0.04	0.11 (0.10–0.17) 0.12 ± 0.02

BPH—patients with benign prostate hyperplasia; PCa surgical—operated patients with adenocarcinoma; PCa palliative—patients with adenocarcinoma not eligible for surgery, who received radiotherapy for bone metastases; CRP—C Reactive Protein; TNFα—tumor necrosis factor α; TNFRI—tumor necrosis factor receptor 1; TNFRII—tumor necrosis factor receptor 2; Fas L—FAS ligand; X ± SD—arithmetical mean ± standard deviation; ^a^—*p* < 0.001 vs. BPH; ^b^—*p* < 0.05 vs. BPH; ^c^—*p* < 0.001 vs. PCa palliative (Kruskal–Wallis test).

**Table 6:** Comparison of the concentration of selected apoptotic markers in the patients with BPH and prostate cancer after prostatectomy procedures and palliative radiotherapy.

Parameter	BPH Median (Range) X ± SD	PCa Surgical Median (Range) X ± SD	PCa Palliative Median (Range) X ± SD
TRIAL	110.86 (95.86–121.57) 180.89 ± 8.55	110.14 (83.00–1153.00) 160.25 ± 209.98	110.14 (87.29–240.14) 118.71 ± 37.99
Caspase 8	0.113 (0.105–0.123) 0.114 ± 0.005	0.109 ^a^ (0.105–0.119) 0.111 ± 0.004	0.112 (0.104–0.134) 0.114 ± 0.007
Caspase 9	5.48 (4.14–9.54) 6.07 ± 1.56	5.90 (4.46–47.94) 7.54 ± 8.45	5.96 (4.67–9.33) 6.37 ± 1.50
cfDNA	828.0 (668.0–1564.0) 855.8 ± 180.2	864.5 (512.0–1248.0) 863.0 ± 131.0	787.0 (602.0–1450.0) 880.6 ± 275.3
met-DNA	73.50 (61.77–79.74) 72.08 ± 5.22	69.23 (51.91–81.50) 69.09 ± 5.77	66.47 ^a^ (57.87–85.58) 67.73 ± 6.52

BPH—patients with benign prostate hyperplasia; PCa surgical—operated patients with adenocarcinoma; PCa palliative—patients with adenocarcinoma not eligible for surgery, who received radiotherapy for bone metastases; CRP—C Reactive Protein; X ± SD—arithmetical mean ± standard deviation; ^a^—*p* < 0.05 vs. BPH (Kruskal–Wallis test).

**Table 7:** Correlation between prostate volume and selected parameters in the patients with BPH and prostate cancer after prostatectomy procedures and palliative radiotherapy.

Parameter	BPH	PCa Surgical	PCa Palliative
Gleason score	-	r_s_ = −0.099 *p* = 0.572	r_s_ = 0.402 *p* = 0.088
Pathological stage	-	r_s_ = −0.194 *p* = 0.263	r_s_ = 0.101 *p* = 0.708
Ki67	r_s_ = 0.323 *p* = 0.062	r_s_ = −0.147 *p* = 0.398	r_s_ = 0.426 *p* = 0.069
PSA	r_s_ = 0.140 *p* = 0.429	r_s_ = 0.089 *p* = 0.613	r_s_ = −0.020 *p* = 0.936
CRP	r_s_ = −0.287 *p* = 0.100	r_s_ = 0.199 *p* = 0.274	r_s_ = −0.163 *p* = 0.578
P-selectins	r_s_ = 0.204 *p* = 0.247	r_s_ = −0.021 *p* = 0.910	r_s_ = −0.163 *p* = 0.579
TNFα	r_s_ = 0.515 *p* = 0.041	r_s_ = −0.056 *p* = 0.789	r_s_ = −0.336 *p* = 0.241
TNFRI	r_s_ = −0.416 *p* = 0.108	r_s_ = 0.360 *p* = 0.077	r_s_ = −0.232 *p* = 0.424
TNFRII	r_s_ = 0.305 *p* = 0.251	r_s_ = 0.126 *p* = 0.548	r_s_ = −0.445 *p* = 0.110
Fas L	r_s_ = −0.048 *p* = 0.861	r_s_ = −0.165 *p* = 0.431	r_s_ = −0.267 *p* = 0.357

BPH—patients with benign prostate hyperplasia; PCa surgical—operated patients with adenocarcinoma; PCa palliative—patients with adenocarcinoma not eligible for surgery, who received radiotherapy for bone metastases; CRP—C Reactive Protein; TNFα—tumor necrosis factor α; TNFRI—tumor necrosis factor receptor 1; TNFRII—tumor necrosis factor receptor 2; Fas L—FAS ligand; r_s_—Spearman’s rank correlation coefficient; *p*—statistical significance (Spearman rang correlation).

## Data Availability

The data presented in this study are available on request from the corresponding author. The data are not publicly available due to patient confidentiality.
